# Pediatric acute-onset neuropsychiatric syndrome and pediatric autoimmune neuropsychiatric disorder associated with streptococcal infections: a delphi study and consensus document about definition, diagnostic criteria, treatment and follow-up

**DOI:** 10.3389/fimmu.2024.1420663

**Published:** 2024-10-24

**Authors:** Roberto Grandinetti, Nicole Mussi, Simone Pilloni, Greta Ramundo, Angela Miniaci, Emanuela Turco, Benedetta Piccolo, Maria Elena Capra, Roberta Forestiero, Serena Laudisio, Giovanni Boscarino, Laura Pedretti, Martina Menoni, Giuditta Pellino, Silvia Tagliani, Andrea Bergomi, Francesco Antodaro, Maria Cristina Cantù, Maria Teresa Bersini, Sandra Mari, Franco Mazzini, Giacomo Biasucci, Agnese Suppiej, Susanna Esposito

**Affiliations:** ^1^ Pediatric Clinic, University Hospital, Department of Medicine and Surgery, University of Parma, Parma, Italy; ^2^ Pediatric Clinic, IRCCS Azienda Ospedaliera Universitaria di Bologna, Bologna, Italy; ^3^ Pediatrics and Neonatology Unit, Department of Medicine and Surgery, University of Parma, Guglielmo da Saliceto Hospital, Piacenza, Italy; ^4^ Pediatric Clinic, University of Ferrara, Ferrara, Italy; ^5^ Primary Care Pediatricians, Azienda Unit Sanitaria Locale (AUSL) Modena, Modena, Italy; ^6^ Primary Care Pediatricians, Azienda Unit Sanitaria Locale (AUSL) Parma, Parma, Italy; ^7^ Primary Care Pediatricians, Azienda Unit Sanitaria Locale (AUSL) Romagna, Forlì-Cesena, Italy

**Keywords:** group A streptococcus, neuroinflammation, obsessive-compulsive disorder, pandas, pans, tics

## Abstract

Pediatric Autoimmune Neuropsychiatric Disorder Associated with Streptococcal Infections (PANDAS) and Pediatric Acute-onset Neuropsychiatric Syndrome (PANS) are broad diagnoses that encompass a range of sudden-onset neuropsychiatric symptoms in children, which can include obsessive-compulsive disorder (OCD), tics, anxiety, emotional instability, and cognitive difficulties. Unlike PANDAS, PANS is not strictly linked to group A streptococcal infections but can be triggered by various infectious or environmental factors. Lights and shadows remain upon the management of children with PANS and PANDAS and there is no clear consensus regarding definition, diagnostic criteria, treatment, and follow-up. The aim of the present study was to evaluate the level of agreement on PANS and PANDAS definition, diagnostic criteria, treatment and follow-up and to assess on the basis of recent studies whether there is a need to modify the current recommendations used by primary care pediatricians and hospital pediatricians in clinical practice in order to improve outcomes. Using the Delphi method, this consensus provides shared indications on PANS and PANDAS management in pediatric age, based on the most updated literature. This work represents, in our opinion, the most complete and up-to-date information on the diagnosis of PANS and PANDAS, as well as consensus statements about several aspects of clinical care. Undoubtedly, more randomized and controlled trials are needed in the pediatric population to better define the best management, also in terms of adequate follow-up examinations and period of observation.

## 1 Background

Group A *Streptococcus* (GAS) is a Gram-positive bacterium that has been associated with asymptomatic infection in the human host, but it can also cause pharyngitis, pyoderma, scarlet fever, or major invasive disease (i.e., septicemia, toxic shock-like syndrome, and necrotizing fasciitis), with the possibility of triggering post-infection immune sequelae (1). In 1998, Swedo et al. described 50 cases of a new pediatric disease called PANDAS (Pediatric Autoimmune Neuropsychiatric Disorder Associated with Streptococcal Infections) (2). PANDAS is defined by the abrupt onset of obsessive-compulsive disorder (OCD), tics and/or food restriction with at least two other debilitating neuropsychiatric symptoms such as emotional changes, personality changes or deterioration in school performance, sensory or motor abnormalities, somatic symptoms or sleep abnormalities and enuresis (3, 4). The disorder often follows a relapsing-remitting course, with neuropsychiatric flares and remissions. There is a lack of epidemiological data on the incidence and prevalence of PANDAS. It is a pre-pubertal disorder with a very young age of onset. Children are expected to show symptoms at an early age, 6.3 years for tics and 7.4 years for OCD (5). Males appear to be more susceptible than females (5). Over time, it has become clear that the diagnosis of PANDAS is underestimated due to the difficulty in demonstrating and documenting the relationship between the onset/recurrence of symptoms and GAS infection (3). On the other hand, the latency period between GAS infection and the onset of PANDAS symptoms is not well defined. Furthermore, some researchers observed that neuropsychiatric manifestations similar to that reported in PANDAS have been described in the context of other infections or diseases (6–8). Thus, the classification of PANS (Pediatric Acute-onset Neuropsychiatric Syndrome) was implemented by considering PANDAS as a subcategory of PANS. In addition, they separated the PANS of infectious origin from non-infectious PANS, introducing a new hierarchy (9).

The exact pathophysiology of PANS is not fully understood. The main hypothesis has been based on molecular mimicry phenomena: antibodies produced against pathogens find a target in brain proteins (10). A recent study supports this hypothesis, identifying cerebral microstructural abnormalities in the thalamus, basal ganglia, and amygdala in children with PANDAS, similar to Sydenham’s chorea (11). Recently, a cross-sectional study described the immunological characterization in an Italian PANDAS cohort: twenty-six PANDAS patients were compared with 11 controls with recurrent pharyngotonsillitis and showed higher tumor necrosis factor (TNF)-alpha and interleukin (IL)-17 levels with lower C3 levels compared to the control group (12). Despite the studies conducted, lights and shadows remain upon the management of children with PANS or PANDAS and there is no clear consensus regarding definition, diagnostic criteria, treatment, and follow-up (13). The aim of the present study was to evaluate the level of agreement on PANS and PANDAS definition, diagnostic criteria, treatment and follow-up in Emilia-Romagna Region, Italy, and to assess on the basis of recent studies whether there is a need to modify the current recommendations used by primary care pediatricians and hospital pediatricians in clinical practice in order to improve outcomes.

## 2 Methods

Our aim to produce a global expert consensus document on the management of PANS and PANDAS was achieved using the Delphi method. This method is a systematic process of forecasting using the collective opinion of panel members, involving a series of discussion sessions to assess expert opinion on controversial topics, based on the published literature, in order to reach a final consensus. Questions are circulated to a group of experts who provide anonymous, iterative and indirect feedback (14, 15).

A panel of pediatricians was responsible for the design and monitoring of the entire study. A committee of experts in primary care pediatrics, pediatric neuropsychiatry, immunologists, rheumatology and pediatric infectious diseases was then selected by the committee on the basis of their research skills and clinical experience. The members were also directors of pediatric units in various hospitals in Emilia-Romagna region, pediatric neurologists and primary care pediatricians representative of the entire regional territory. The project coordinators and the scientific committee developed a questionnaire with 76 questions focusing on 4 main topics selected on the basis of available literature and clinical experience: (1) Epidemiology (prevalence and incidence of disease, etiology); (2) Diagnostic criteria (clinical and laboratory parameters); (3) Therapy (first-line therapy); (4) Follow-up (frequency of visits, tests to be performed). The statements were proposed after a careful review of the current scientific literature, including original research, systematic reviews and reviews, meta-analyses, recommendations and guidelines, identified using PubMed and Medline. For each question the literature, including the references, was presented to the expert panel.

Following the Delphi method, statements were uploaded to a dedicated web platform and voted on by the panel of experts using a five-point Likert scale (1 = strongly disagree, 2 = somewhat disagree, 3 = neutral, 4 = somewhat agree, 5 = strongly agree). Questions for which less than 75% of the responses were 4 and 5 were considered uncertain.

The first round of the survey was blinded to other panel members. Experts responded within one month using the online survey application Google Forms. The responses from the first round were collated and analyzed by an independent statistician, after which we held a virtual meeting to discuss the outstanding issues, where the responses and evidence were presented. Clarifications, adjustments and refinements were made to the indications and appropriateness scores. Consensus was reached when, for each statement, scores 4 and 5 represented at least 75% of the votes. Once consensus was reached for each statement, the authors revised and wrote the final paper. In the end, 37 statements emerged from the original 76 questions. Data analysis was performed using Stata 11 (StataCorp, College Station, TX, USA) and graphical data processing and presentation using Microsoft Excel.

## 3 Results

### 3.1 Epidemiology

**Statement 1.** *PANDAS is a term used to describe populations of children in whom symptoms of obsessive-compulsive disorder (OCD), tics or other neuropsychiatric disorders are exacerbated by GAS infection. PANDAS is considered a subset of the PANS category.*

In the 1980s, researchers at the National Institutes of Health (NIH) identified a subgroup of children with OCD who presented with a sudden onset of their usual psychiatric symptoms, typically following an infection caused by a variety of pathogens (4). These disorders were originally defined as “pediatric infection-triggered, autoimmune, neuropsychiatric disorders” (PITANDS). Subsequent studies focused on a subset of cases triggered by GAS, hypothesizing a pathogenic mechanism similar to that leading to the development of Sydenham’s chorea (SC) (5). This led to the identification of patients with OCD whose symptoms appeared to be triggered by GAS infections. The acronym PANDAS was coined to refer to “pediatric autoimmune neuropsychiatric disorder associated with streptococcal infections” (2, 5).

After the initial association between PANDAS and GAS, studies have shown that GAS is not the only pathogen associated with the acute neuropsychiatric syndrome. It is hypothesized that PANS is much broad than PANDAS and includes not only disorders that may be associated with a previous infection, but also acute neuropsychiatric disorders without an apparent infectious trigger (3). The acute onset of OCD or tics is often accompanied by other symptoms such as mood changes, separation anxiety, sleep disturbances (i.e., difficulty falling asleep, frequent night wakings, bedwetting), irritability, hyperkinesia, and worsening school performance. The disease usually has a relapsing-remitting course, with neuropsychiatric relapses or exacerbations (‘flare’) alternating with periods of remission (16, 17).

Overall, all the text in this statement obtained sufficient agreement during the first survey and was confirmed in the second survey.

**Statement 2.** *Epidemiologically, the true incidence and prevalence of PANS and PANDAS are not known. The male gender seems to be more affected. Tics typically appear at an average age of about 6 years and OCD symptoms at about 7 years. In any case, children between the ages of 5 and 12 years would be more susceptible to developing PANDAS because of their frequent exposure to GAS and their tendency to respond to infections with more antibodies than adolescents and adults.*

There is a lack of epidemiological data regarding the incidence, prevalence, and demographic characteristics of PANS and PANDAS. PANDAS has been considered a controversial diagnosis due to conflicting findings in various immunological and epidemiological studies indicating potential underdiagnosis (3, 18). Notably, in PANDAS males appear to exhibit a higher susceptibility. Since the first description of 50 cases affected by PANDAS in 1998, the male gender appeared to be a risk factor (¾ of the subjects were males). Male predominance has been reported for both tic disorders and obsessive-compulsive disorders that emerged in early childhood (5). The mechanism behind this males’ increased vulnerability is unknown. A cross-sectional study, recently published, revealed a male-to-female ratio of 3.33:1, confirming a higher prevalence of the disease in males (12). Similarly, a systematic review published in 2022 confirmed a statistically significant difference in male prevalence (59.9% males) (19). These results were further supported by a survey of PANDAS conducted by Calaprice et al. (male predominance 65%) (20).

Regarding age, the prepubertal period seems to be the most affected, with a very young age of onset. Thus, it is expected that children would experience symptoms at an early age, 6.3 years for tics and 7.4 years for obsessive-compulsive symptoms (5). Children between the ages of 5 and 12 years would be more likely to develop PANDAS due to frequent exposure to GAS (21). It has been reported that children could result in asymptomatic GAS infection (21). Recently, a prospective, longitudinal, multi-cohort study was done in response to a rise in scarlet fever notifications in England (22). Using genome sequencing to confirm common sources of transmission, authors described a high prevalence of the outbreak strain among asymptomatic classroom contacts. Despite treatment and implementation of standard hygiene measures, transmission was observed underlining a need for improvement of case management (22). During COVID-19 pandemic it has been described that the immune response to pathogens in younger children is more robust than in adolescents and adults, considering the role of trained immunity (23). This evidence could lead us to think that the greater antibody response of children in this age group and the repeated exposure to the pathogen may make them more at risk of developing PANDAS (24). However, a case of PANDAS in an adult has been described, which could challenge the exclusively pediatric etiology of the pathology (25). Further studies are needed to confirm these hypotheses, especially large-scale studies to characterize the incidence, prevalence and demographic characteristics of PANDAS.

Overall, most of the text in this statement obtained sufficient agreement during the first survey. Uncertainty was initially observed about the incidence and prevalence of PANDAS according to gender and the average age of onset. After review of recent literature and collective discussion on available epidemiological data, panelists agreed on defining the prevalence higher in the male gender, with an average onset of symptoms at the age of 6-7 years.

**Statement 3. A** *genetic predisposition to PANDAS development is suspected, although genetic testing is not currently required for diagnosis.*

A complete medical and psychiatric history of the patient should be taken and correlated with the family history for a possible genetic evaluation. One of the fascinating things about PANS and PANDAS is that they “come out of the blue” and affect a previously healthy, neurodevelopmentally normal (or more often, highly intelligent) child. Indeed, a recent study reported the results of whole exome sequencing performed on a US cohort of 386 cases and 10 European cases suffering from a severe form of PANS (26). Mutations were found in 11 genes (*PPM1D, SGCE, PLCG2, NLRC4, CACNA1B, SHANK3, CHK2, GRIN2A, RAG1, GABRG2* and *SYNGAP1*). These genes fall into two functional categories: one regulates peripheral immune responses and microglia, the other is mainly expressed in neuronal synapses. Mutations in the same neuronal genes have also been described in autism spectrum disorders and myoclonus-dystonia. Indeed, in 50% of cases the development of PANS overlapped with a pre-existing neurodevelopmental disorder, indicating a probable genetic predisposition (26). Overall, all the text in this statement obtained sufficient agreement during the first survey and was confirmed in the second survey.

**Statement 4.** *PANDAS seems to be more common among children with a family history of rheumatic fever.*

Familial factors might contribute to the pathogenesis of PANDAS. In children diagnosed with PANDAS, first-degree relatives have been observed to have increased rates of obsessive-compulsive disorder, tic disorders, and acute rheumatic fever, suggesting that children may have inherited a specific vulnerability to non-pyogenic post-streptococcal sequelae (2). A study conducted by Calaprice et al. described the association between autoimmune diseases and relatives of patients diagnosed with PANDAS, revealing an elevated rate of familial (particularly maternal) autoimmunity and rheumatic fever, and low rates of familial neuropsychiatric pathology (20). Autoimmune conditions were common among patient’s first-degree relatives, especially mothers, with 20% reported to have at least one serious autoimmune diagnosis (such as multiple sclerosis, Crohn’s disease, PANS, lupus, rheumatoid arthritis, hypothyroidism and/or hyperthyroidism). The rheumatic fever was described in 3% of mothers and 1% of fathers, and 14% of patients had at least one grandparent with this diagnosis (20). This was confirmed by the cross-sectional study mentioned before, which described, within the PANDAS cohort, three patients with a positive family history of rheumatic fever among first-degree relatives, suggesting a potential role of genetic susceptibility in the development of autoimmune sequelae following GAS infection (12).

Uncertainty was initially observed about the family history correlation. After review of recent literature and collective discussion on available data, panelists agreed on considering the family history an important element during the anamnesis.

***Statement 5.*** *It is supposed that a previous GAS infection could act as a PANDAS trigger, due to molecular mimicry mechanisms, causing an abnormal immune response. Moreover, a role of increased oxidative stress and neuroinflammation dysregulation caused by gut microbiota, has been proposed. For these reasons, immunomodulatory therapy could be considered in selected cases.*

The most studied theory for PANDAS pathogenesis is post-streptococcal autoimmunity. GAS infection and the subsequent generation of anti-streptococcal antibodies are thought to activate an autoimmune response in susceptible hosts by ‘molecular mimicry’ of basal ganglia epitopes, owing structural similarities between the streptococcal antigens and antigens of the basal ganglia (10, 27–30). Specific antibodies against the human caudate nucleus and anti-neuronal antibodies have been observed in children with OCD and PANDAS (30–32). This pathogenetic model is based on clinical similarities to Sydenham’s chorea. In fact, also in patient affected by Sydenham’s chorea the basal ganglia are damaged (2, 33).

Moreover, a recent study described cerebral microstructural anomalies in children with PANDAS, specifically in thalamus, basal ganglia, and amygdala, studying brain structures with diffusion-weighted MRI (11, 31). Specifically, selective neuroanatomic differences in putamen and significant increases in size confirmed the hypothesis of a cross-reactive antibody-mediated inflammation of the putamen as being the pathophysiologic mechanism for this disorder (28).

In the context of PANDAS, D1R autoantibodies were identified in a study by Cox et al. (34), highlighting the role of dopamine receptor autoantibodies in this disorder. The importance of dopamine receptor autoantibodies in Sydenham’s chorea is further detailed by Cox et al., who showed that the autoantibodies from Sydenham’s chorea were expressed in transgenic mice and found to target the basal ganglia, including the substantia nigra and the ventral tegmental area (35). Autoantibodies in Sydenham’s chorea primarily target the D2 receptor, contributing to symptoms of movement and chorea (35). The ratio of D1R/D2R receptors is directly associated with symptom severity, as demonstrated in the study by Ben-Pazi et al. (36). In a recent study, the first human monoclonal antibodies derived from PANDAS were identified to be against the D1R with signaling of the D1R (37). Specific epitopes of D1R and D2R extracellular loops were identified in the two diseases. The study separated PANDAS/PANS from Sydenham’s chorea based on the D1R vs D2R autoantibodies (37).

In addition to the auto-immunity hypothesis, a possible role of immunodeficiency/deregulation has been reported (38). A cross-sectional study comparing 26 PANDAS patients with 11 controls, analyzed the correlation of immunological parameters with clinical-anamnestic data (12). No inborn errors of immunity were detected in either group. However, a trend toward higher TNF-alpha and IL-17 levels, and lower C3 levels, was detected in the PANDAS patients compared to the control group. Maternal autoimmune diseases were described in 53.3% of PANDAS patients suggesting a possible correlation with enduring inflammation and auto-immunity (12). Recent studies have suggested that alterations in the C4 gene may contribute to the pathophysiology of PANDAS. Sekar et al. demonstrated that individuals with certain neuropsychiatric conditions, including PANDAS, often exhibit reduced C4A expression (39). This reduction may lead to an impaired clearance of immune complexes and apoptotic cells, resulting in an increased inflammatory response within the brain. Further supporting this notion, another study examined the genetic profiles of children with PANDAS and found a significant correlation between reduced C4A gene expression and the severity of neuropsychiatric symptoms (40). This finding suggests that alterations in the C4 gene may influence the clinical presentation and progression of PANDAS, potentially through mechanisms involving dysregulated immune responses and chronic inflammation. Furthermore, other studies demonstrated that children with PANDAS often exhibit variations in the number of C4 gene copies, particularly showing a reduction in C4A gene expression (39). This genetic alteration may exacerbate the autoimmune response triggered by streptococcal infections, thereby worsening the clinical outcomes in affected children. Additionally, the altered complement activity due to C4 gene variations may lead to inappropriate synaptic pruning during critical periods of brain development (41). This mechanism could underlie the neuropsychiatric manifestations observed in PANDAS, as excessive synaptic pruning has been implicated in various neurodevelopmental and psychiatric disorders.

Recent researches have also emphasized the possible role of gut composition: streptococcal infections could modify gut bacterial communities, resulting in a pro-inflammatory state by favoring specific bacterial strains linked to gut inflammation and the activation of the auto-immune response (42).

Some studies report benefits of immunomodulatory therapy, such as IVIGs or plasma exchange (43–48) compared to placebo, which had no effect on OCD and tics (49). Furthermore, not all studies confirmed the findings of autoantibodies against the basal ganglia in children with PANDAS (50).

Overall, most of the text in this statement obtained sufficient agreement during the first survey. Uncertainty was initially observed on the role of increased oxidative stress and neuroinflammation dysregulation caused by gut microbiota as well as on the efficacy of immunotherapy due to the lack of robust data. After review of recent literature and collective discussion on available data, panelists agreed with the postulated etiologies implied in PANS and PANDAS onset.

### 3.2 Diagnostic criteria

**Statement 6.** *PANDAS is defined by: 1) the presence of OCD and/or tic disorders; 2) onset in childhood between the age of 3 and the onset of puberty; 3) a clinical course characterized by sudden onset and dramatic worsening of symptoms; 4) the temporal relationship between GAS infection and onset/recurrence of symptoms; 5) the presence of neurological symptoms on physical examination during a relapse, such as motor hyperactivity (difficulty staying seated, restlessness) and choreiform movements, especially when aggravated by forced postures.*

Over time, it emerged that the diagnosis of PANDAS was limited to cases in which the correlation between the onset/recurrence of symptoms and GAS infection was evident (21). Thereafter, new diagnostic criteria were formulated to include patients meeting all PANDAS criteria except for the association with GAS infection. The acronym PANS (Pediatric Acute-Onset Neuropsychiatric Syndrome) was proposed to make a more inclusive diagnosis (9). In addition, the presence of tics and prepubertal onset were no longer required while the acute onset of OCD and restrictive eating disorder have been emphasized (9).

Overall, all the text in this statement obtained sufficient agreement during the first survey and was confirmed in the second survey.

**Statement 7.** *PANS diagnostic criteria are sudden and dramatic onset of OCD and/or restrictive food intake (<48 hours) associated with concomitant presence of additional neuropsychiatric symptoms. The OCD symptoms must be severe and frequent. Additional neuropsychiatric symptoms should be at least two of the following: 1) anxiety; 2) emotional instability and/or depression; 3) irritability, aggressiveness and/or severe oppositional behaviors; 4) emotional regression; 5) deterioration of school performance (related to symptoms similar to attention deficit/hyperactivity disorder, memory deficits, cognitive changes); 6) sensory or motor abnormalities; 7) somatic signs and symptoms, including sleep disorders, enuresis or urinary frequency. Symptoms should not be explained by a known neurological or medical disorder, such as Sydenham’s chorea.*

To diagnose PANS, it is necessary to document the sudden and acute onset (within 72 hours) of an OCD or the appearance of a restrictive eating disorder (16). Gromark et al. presented a systematic evaluation of 45 patients included in a Swedish cohort (51). They reported in most cases the abrupt onset of OCD and/or anorexia, emotional lability and a wide range of somatic symptoms. The severity of symptoms may lead to significant loss of function and drastic deterioration of quality of life (51). Food restriction has been reported to occur in the context of obsessive fears of contamination as well as in the context of the sudden onset of fears of swallowing, choking, or vomiting that are often associated with sensory phenomena (i.e., the perceived texture or appearance of food). In rare cases, these fears may lead to the child refusing to take anything orally, including liquids. Fear of contamination may lead to dietary restrictions on all or some foods. Body image distortion may also be present (52).

Among neuropsychiatric symptoms, the most common onset symptoms reported are OCD (89%), anxiety (78%) and emotional lability (71%) (51). Children may experience auditory or visual hallucinations as well as violent imagery and suicidal or homicidal ideation. Motor and phonic tics (i.e., whooping, wringing and twisting hands, convulsing) are common; also behavioral regression and extreme compulsions (licking shoes, barking) were sometimes shown (52). During an acute episode, parents reported that the child may appear hyperalert, frightened or depressed; agitation, irritability, and episodes of aggressivity were also common. Emotional lability (emotional incontinence) is a hallmark symptom of PANS and is characterized by involuntary and uncontrollable episodes of crying or laughing that are often mood-incongruent (i.e. laughing uncontrollably when angry or sad) (4). Speech is often affected, with a variety of manifestations such as ‘‘baby talk’’ secondary to developmental regression, a paucity of speech, mutism, or new onset of stuttering (4). Prosell et al. reported that in a group of Swedish children with diagnosed or suspected PANS or PANDAS majority of the participants (54.5%) had changes in speech fluency with higher speech rate, superfluous verbal use, stuttering, reduced intelligibility, reduced speech production and reduced vocabulary, trouble finding the right word (53). Memory disorders are also part of the PANDAS syndrome: children often cannot remember the details of their symptoms or their impact on functioning (4). Depression is also common, especially during later stages of the illness (4).

PANS-related signs that might be present on physical examination include dehydration or emaciation secondary to restricted intake of fluids or food, sequelae of compulsive behaviors like a red ring around the mouth from excessive lip-licking or chapped hands from excessive washing (4). Moreover, they can present motor and/or phonic tics, choreiform movements or signs of GAS infection (i.e., pharyngitis), so clinical evaluation is fundamental (4).

Overall, all the text in this statement obtained sufficient agreement during the first survey and was confirmed in the second survey.

**Statement 8.** *The diagnosis of onset or exacerbation of PANDAS requires evidence of a close temporal association with GAS infection, although the time window is not yet well defined.*

PANDAS is strictly related to infections: GAS infection may trigger both onset and exacerbations, through a nonspecific immune activation mechanism (5, 54). Indeed, there is a growing evidence that neuropsychiatric or behavioral disorders such as OCD or Tourette’s syndrome may be at least partly due to a post-infectious autoimmune phenomenon caused by GAS, as shown in a case-control study in which patients diagnosed with OCD, tics or Tourette’s syndrome were more likely than controls to have had a previous streptococcal infection in the three months before the onset of the disorder (55). The risk was even higher in children with multiple streptococcal infections in the previous 12 months (45). This is supported by the increased prevalence of human caudate nucleus antibodies (56) and antineuronal antibodies in OCD and PANDAS patients (28–30, 57, 58) through ‘‘molecular mimicry’’ of basal ganglia epitopes in susceptible hosts, which may contribute to the pathophysiology of motor and behavioral disturbances (4, 20, 34–36). Approximately, 77% of 144 exacerbations appear to be associated with confirmed or suspected GAS infection and patients with a history of multiple GAS infections were found to have more frequent and severe relapses, with a quantitative association (35). Furthermore, high or increasing levels of streptococcal antibodies are associated with a relapsing-remitting course (55, 59).

The exact latency period between GAS infection and the onset of PANDAS symptoms is not well defined. Considering the pattern of Sydenham’s chorea, which is similar to PANDAS in terms of pathogenesis, it has been suggested that the onset of neuropsychiatric symptoms may occur from one to 6-9 months after GAS infection; however, relapses occurring after GAS infection may have a much shorter latency, even as short as days or weeks (60). Moreover, as in the case of Sydenham’s chorea and rheumatic fever, some symptoms recurrences may not be associated with documented GAS infections (61), so the association may be entirely coincidental. An additional issue is the differentiation of a triggering GAS infection (clinical or subclinical) and GAS carrier states (31). The most observed antecedent infection seems to be an upper respiratory infection, including rhinosinusitis, pharyngitis, or bronchitis. It is not yet clear if any of those three presentations is more likely than the others to be associated with the initiation of PANS (4, 20).

Overall, all the text in this statement obtained sufficient agreement during the first survey and was confirmed in the second survey.

**Statement 9.** *Aetiological agents implicated in the development of PANS include varicella virus, Epstein-Barr virus, herpes simplex virus, Mycoplasma pneumoniae, Enterovirus, Chlamydia pneumoniae, Borrelia burgdorferi, Toxoplasma gondii, and SARS-CoV-2. The most common clinical manifestation related to PANS onset seems to be an upper respiratory tract infection, including rhinosinusitis or pharyngitis*.

Several types of infections have been described in PANS, including upper respiratory tract infections, such as rhinitis, pharyngitis and tonsillitis, as well as gastrointestinal, dental and skin infections (2). It has been hypothesized that aetiological agents different to GAS, especially viruses, could induce OCD, tics and other neuropsychiatric disorders. These include varicella zoster virus (VZV), Epstein-Barr virus, herpes simplex virus, Enterovirus, and HIV (6, 62, 63). Among bacterial and protozoal infections, *Mycoplasma pneumoniae*, *Chlamydia pneumoniae*, *Borrelia burgdorferi* and *Toxoplasma gondii* have been associated with PANS development (6, 62, 63). As with GAS infection, infections induce the process of autoimmunity: it has been proposed that similar molecular structures between microorganisms and native molecules can induce a cross-reaction with the generation of autoantibodies targeting endogenous structures (64, 65).

Dahiya et al. described two cases of neuropsychiatric symptoms following VZV infection, with evidence of central nervous system (CNS) inflammation following the resolution of infection (7). Two children (6-year-old male and 10-year-old female) developed neuropsychiatric syndrome after 3–6 weeks of a confirmed VZV infection with intrathecal oligoclonal bands (i.e., immunoglobulins). They presented cognitive difficulties, seizures, agitation, insomnia, personality changes, stereotyped hand movement, psychiatric symptoms (such as anxiety, tearfulness, fearfulness, emotional dysregulation, anguish, uncontrollable crying) with an onset of 3-6 weeks after confirmed VZV infections. Neuroleptics and sedatives resulted in only a mild reduction of the symptoms and IVIGs were also unsuccessful. Interestingly, both cases were very responsive to steroid therapy, confirming the immunological nature of the symptoms due to cross-reactivity of anti- VZV antibodies and the brain. They proposed the term VANS (Varicella-Associated Neuropsychiatric Syndromes) to classify these syndromes (7).

Efe reported a case of twin adolescents with COVID-19-associated PANS (8). Dizygotic twin sisters presented abrupt and acute onset of severely restrictive food intake, weight loss, OCD, anxiety with intermittent auditory and visual hallucinations, depression, attention deficit and sleep disturbances, simultaneously accompanied by milder neurologic symptoms after SARS-CoV-2 infection validated with laboratory testing and negative biomarkers for other possible bacterial or viral agents. They also showed generalized epileptic anomaly and a vermian/folial atrophy in the cerebellum. The twins were treated with psychotropic agents, antibiotics and antiepileptic. After these treatments, IVIGs transfusion was required showing resolution of psychiatric symptoms (8). Recently, a case series of 10 pediatric patients with acute onset or relapse of PANS symptoms after SARS-CoV-2 infection has been published (66). The patients presented neuropsychiatric-PANS symptoms triggered by SARS-CoV-2 infection; they benefited from corticosteroids for both global clinical severity and global functioning. The authors’ data confirmed that the neuropsychological sequelae of SARS-CoV-2 overlap with those reported in PANDAS and PANS (66). These data also suggested that treatment with oral prednisone is associated with symptoms resolution (66). Despite being interesting, all these novelties are reported by single case series, so further studies are required to confirm these hypotheses in the pediatric population.

Overall, most of the text in this statement obtained sufficient agreement during the first survey. Uncertainty was initially observed about the correlation with SARS-CoV-2 infection as trigger event of PANS. After review of recent literature and collective discussion on available epidemiological data, panelists agreed on an implication of this infection in the pathogenesis of PANS, even if more studies are needed to ascertain this conclusion.

**Statement 10.** *The etiology of PANS is not necessarily infectious.*

Although relapses were often associated with infections, some patients have been reported to experience PANS flares in response to a variety of environmental exposures, including certain foods, additives, and allergens (20). The association with pre-existing diseases or conditions commonly thought to have an autoimmune or inflammatory etiology has been reported. The most common is severe asthma, but also severe atopic eczema, multiple food allergies, celiac disease, post-infectious arthritis and Henoch-Schonlein purpura have been implied (51).

Overall, all the text in this statement obtained sufficient agreement during the first survey and was confirmed in the second survey.

**Statement 11.** *The diagnosis of GAS infection in patients with PANDAS is made when a throat or skin swab (rapid antigen test or culture) is positive with high levels of antistreptolysin O/antistreptolysin titer (ASO) and anti-DNase B (ADB) antibodies.*

GAS infection must be confirmed by throat culture or rapid antigen test, or by testing other symptomatic sites such as the nasal cavity, skin, perianal or vaginal areas (21). It is useful to investigate whether there has been a history of scarlet fever rash, impetigo, perianal or vulvar dermatitis, or deep tissue GAS infection within the last 6 months. There are also anti-GAS antibodies, such as antistreptolysin O (ASO) and anti-deoxyribonuclease B (anti-DNase B), which increase in serum after a GAS infection. ASO/TAS antibodies peak from three to five weeks after GAS pharyngitis, whereas ADB antibodies peak from six to eight weeks after GAS pharyngitis (21).

In some individuals, cross-reactivity leads to direct or immune complex-mediated damage, involving kidney antigens (poststreptococcal glomerulonephritis), heart antigens (rheumatic fever) or brain antigens (Sydenham’s chorea) (59, 67). Increases in GAS antibodies observed within a few weeks from the onset of OCD or tics are not sufficient to establish a diagnosis of PANDAS; however, they may support a diagnostic suspicion. Anti-streptococcal antibody titres can remain elevated for several months, so studies have observed that seropositivity can be associated with symptom exacerbations, but also that seronegativity (or a decrease in titers) is associated with symptom remission (21). A longitudinal study of a small number of children with PANDAS suggests that relapses are associated with a rapid and dramatic (doubling or more) increase in anti-streptococcal titers, whereas symptom remission and decreases in titers occur more slowly (59). The kinetics of ASO/TAS or ADB antibodies are highly variable depending on the site of the streptococcal infection (2, 68).

Overall, most of the text in this statement obtained sufficient agreement during the first survey. Uncertainty was initially observed about the timing and the execution of the optimal laboratory method in confirming a GAS infection. After review of recent literature and collective discussion on available data, panelists agreed on the statements above-mentioned.

**Statement 12.** *Diagnosis of PANS and PANDAS is a diagnosis of exclusion.*

PANS and PANDAS symptoms overlap with a number of psychiatric disorders, including OCD, Tourette’s syndrome, ADHD, depression, and bipolar disorder (21). They are also similar to symptoms that may be present in autoimmune diseases, inflammatory diseases, infectious diseases and immunodeficiencies (21). It is therefore important to note that PANS and PANDAS are a “diagnosis of exclusion,” and that other known medical conditions must be ruled out before a diagnosis of PANS or PANDAS can be made (4, 20).

Behavioral manifestations often lead to rapid referral to psychological or psychiatric services, but all patients should receive a full medical assessment. In some cases, children with PANS or PANDAS present with visual or auditory hallucinations; these cases deserve special attention as the symptoms may appear identical to psychotic symptoms seen in conditions such as schizophrenia, bipolar disorder and cerebral lupus (2). The acute onset of OCD is often accompanied by other symptoms such as mood swings, separation anxiety, sleep disturbances (i.e., difficulty falling asleep, frequent night waking, enuresis), irritability, hyperkinesia and academic decline (2).

Overall, all the text in this statement obtained sufficient agreement during the first survey and was confirmed in the second survey.

**Statement 13.** *There are currently no circulating biomarkers/autoantibodies pathognomonic for PANS or PANDAS*.

In terms of laboratory testing, currently there is no biomarker for the diagnosis of PANS or PANDAS. The Autoimmune Brain panel (previously known as “Cunningham Panel”) has been proposed to measure PANS and PANDAS-associated antibodies, including anti-D1/D2R, anti-β-tubulin, and anti-lysoganglioside antibodies, as well as CaMKII activity (69, 70). Currently, Moleculera Labs (www.moleculera.com) are the only CLIA (Clinical Laboratory Improvement Amendments) certified laboratories that offer testing for anti-neuronal antitubulin, anti-lysoganglioside, and anti-dopaminergic receptor antibody titers by enzyme-linked immunosorbent assay (ELISA), as well as assays to measure CaMKII signaling. Although the Moleculera panel may provide useful auxiliary information for children with suspected PANS and PANDAS, it is not yet clear what the sensitivity and specificity of these tests may be in pediatric patients and their clinical use (71).

The studies in general support the hypothesis that the antineuronal autoantibodies are important in PANS and PANDAS, appearing elevated in children who exhibited symptoms and reduced upon clinical improvement due to immunomodulatory therapy or antibiotic treatment. Work in press will continue to support the hypothesis that PANDAS and Sydenham’s chorea are a dopamine receptor encephalitis with slightly different autoantibodies leading to different symptoms and outcomes (72). However, further research is needed to uncover diagnostic and therapeutic biomarkers that will allow the characterization of individuals with PANS and PANDAS who most likely benefit from immunomodulatory interventions (73).

Interestingly, recent studies showed that the PANDAS children tested positive for D1R autoantibodies, whereas D2R autoantibodies are important in Sydenham’s chorea (34–36). Shimasaki et al. defined the PANDAS child and developed an algorithm to support it: PANDAS in general has little to no D2R autoantibodies, whereas D2R autoantibodies are associated with Sydenham’s chorea (74). Recent studies reported peptide extracellular loop epitopes recognized by the D1R and D2R autoantibodies for PANDAS and Sydenham’s chorea (37), and there are animal models that evaluated the role of D2R as antipsychotic drug targets (75).

Overall, all the text in this statement obtained sufficient agreement during the first survey and was confirmed in the second survey.

**Statement 14.** *In patients suffering from PANS or PANDAS it is possible to find alterations in the EEG or neuroimaging investigations. These tests are not part of the diagnostic work-up for PANS and PANDAS but can be useful to exclude other organic pathologies.*

Considering the severity of the symptoms experienced by some patients, further investigations such as magnetic resonance imaging (MRI) and EEG have been performed (21). Brain MRI is useful when other conditions are suspected (i.e., vasculitis, encephalitis) or when the patient has severe headaches, cognitive deterioration or psychosis. In some severe cases, the MRI has shown inflammatory changes, including volumetric changes. A study of 34 patients with PANS showed increased diffusion patterns in all brain regions, particularly the deep grey matter (thalamus, basal ganglia and amygdala) (11). EEG can be helpful in detecting focal or generalized slowing and/or epileptiform activity. These signs of abnormal brain activity or irritability were found in 7 out of 42 (16%) patients with PANDAS (11). However, there is a paucity of data on the use of electrophysiological studies in this population. Further research into the sleep disturbances in patients with PANS or PANDAS is needed. Lumbar puncture should be considered in the presence of MRI or EEG abnormalities, or encephalopathic symptoms such as delirium, altered consciousness, seizures, or psychosis (4). In addition, Positron Emission Tomography (PET) imaging showed possible underlying neuroinflammation in the bilateral caudate nuclei in both PANDAS and Tourette syndrome patients (18).

Overall, all the text in this statement obtained sufficient agreement during the first survey and was confirmed in the second survey.

### 3.3 Therapy

**Statement 15.** *The PANS and PANDAS treatment is based on antibiotics, psychotherapeutic treatment (through drugs and/or cognitive-behavioral therapy) and –on immunomodulatory therapy in severe selected cases.*

The PANS/PANDAS Research Consortium Guidelines have been available since 2017 and they are a useful tool to address the treatment once a proper characterization and diagnosis has been made (9). They address, among others, the following topics: 1) antibiotic treatment and secondary prophylaxis; 2) immunomodulatory therapies based on the severity of symptoms; 3) symptomatic control (i.e., medications which relieve OCD, anxiety, impulsivity, motor tics as well as behavioral interventions that can decrease OCD symptoms and improve functioning at home, school and with peers). The importance of these guidelines is given by the systematic work the participants of the Consortium have done, first reviewing the literature and combining it with the experience they had on more than 1000 children with PANS or PANDAS. Moreover, the importance of this work is also due to its multidisciplinary character since several experts were included (i.e., child psychiatrists, specialists in pediatric infectious diseases, neurologists, microbiologists, immunologists and rheumatologists).

Overall, all the text in this statement obtained sufficient agreement during the first survey and was confirmed in the second survey.

**Statement 16.** *In children with PANS or PANDAS with an identified etiological diagnosis, specific treatment of infectious disease should be recommended. Neuropsychiatric symptoms (especially the obsessive-compulsive ones) improve rapidly with an effective antibiotic treatment in eradicating the infection.*

Specific treatments of infectious diseases causative of PANS and PANDAS should be undertaken as a priority; this could improve both neurological and psychiatric symptoms. In fact, despite the scarcity of studies, in some of them a reduction in neuropsychiatric symptoms after the starting of a specific antimicrobial treatment has been noticed, especially in studies concerning acute GAS infection (55, 76, 77). In a prospective study conducted over a 3-year period, 12 school-aged children with new-onset PANDAS were enrolled: obsessive-compulsive symptoms rapidly disappeared after starting a proper antibiotic course eradicating the GAS infection, even if a certain degree of recurrence was noticed (associated with new GAS infection) (78). However, the statistical sample size was small, which is also true for the other studies mentioned, and cannot lead to final conclusions. By extent, a similar reasoning has been applied to infections caused by other pathogens. So, the PANS/PANDAS Research Consortium suggested anti-infective treatment protocols for both GAS infections and infections caused by other bacteria or viruses (65).

Concerning GAS infections, an initial course with an effective treatment on GAS (amoxicillin, penicillin, cephalosporin in case of documented allergic reactions) for 6-10 days is recommended (79). Concerning non-streptococcal PANS, specific treatment is recommended for *M. pneumoniae* infection, varicella or influenza (46, 78). Antimicrobial treatments for other specific infectious diseases should be tailored on a case-by-case basis, according to the most accepted and widespread society guidelines.

Overall, most of the text in this statement obtained sufficient agreement during the first survey. Uncertainty was initially observed about the role of antibiotics in improving the neuropsychiatric symptoms, but after the review of recent literature and collective discussion on available data, panelists agreed on a plausible role in reducing neuropsychiatric symptoms.

**Statement 17.** *In the case of PANS triggered by viral infections, some patients showed a good response to antivirals and to corticosteroid therapy, but there is little evidence in literature. The use of antibiotics and immunomodulatory agents is not recommended in children who present only an acute psychiatric symptomatology and where an infectious etiology has been excluded.*

In case of viral infections which have led to an acute neuropsychiatric symptomatology, some patients have shown a good response to antivirals and to immunomodulatory therapy with corticosteroids (21). However, data on this issue come only from some case reports with a very low number of patients which had – in some cases – a very disruptive onset symptomatology, so these data are very partial and not generalizable. In particular, a case series of two pediatric patients with VZV infection showed a good response to intravenous corticosteroid therapy after a first-proposed line treatment which consisted in IVIG, risperidone and sedative, with a very prompt response in both cases (7). Another case series led to similar conclusions, although the etiology analyzed was not ascertained for viral infections though the suspicion was very high (46). More systematic studies on this issue would be useful to arrive at meaningful conclusions, so a standardized treatment with corticosteroids cannot be recommended routinely; it should be evaluated on the single clinical case.

Ultimately, when an infectious etiology has been excluded it is not recommended undertaking antibiotic therapy; the few data on immunomodulatory therapy do not recommend it equally, but a discussion on the single case should be taken. However, the PANS/PANDAS Research Consortium proposed a series of laboratory and eventually instrumental investigations that help confirming or excluding and infectious etiology; furthermore, a work-up before pursuing immunomodulatory therapies was recommended, which includes also lumbar puncture, MRI, EEG (with a sleep study, if possible) in addition to a correct assessment of immunodeficiencies (autoimmunity defects are more common, alongside with a greater susceptibility to infections, which could worsen under an immunomodulatory treatment) (78). Differently to our position, the PANS/PANDAS Research Consortium also suggested a trial of antibiotics, even when there is no obvious source of bacterial infection (78).

Overall, most of the text in this statement obtained sufficient agreement during the first survey. Uncertainty was initially observed about the role of corticosteroids because of the scarcity of literature data, but after a collective review and collective discussion on available data, panelists agreed on a plausible role.

**Statement 18.** *Antibiotic therapies in children with PANDAS are effective in eradicating the causative pathogen during the active phase of the infection, but further evidence is needed to determine its effectiveness in terms of prophylaxis. There are no studies identifying an appropriate dosage for a possible prophylaxis schedule in patients with PANDAS: a possible antibiotic prophylaxis scheme could be represented by intramuscular penicillin benzathine 600,000 IU/month (1,200,000 IU/month for weight > 27 kg), similarly to the schemes used in the prevention of acute articular rheumatism. Another possible scheme could be oral amoxicillin (25 mg/kg once daily). The duration of one of the antibiotic prophylaxis schemes remains unknown. Antibiotic prophylaxis should be stopped if the neuropsychiatric symptoms do not improve or in case the child has a recurrence of neuropsychiatric symptoms while receiving antibiotic prophylaxis.*

While an important role of antibiotic therapy in the acute phases of PANDAS and an emerging role in PANS has been achieved (78), still little can be concluded about a possible prophylaxis schedule in terms of antimicrobial agent that should be used and duration of treatment. This statement is particularly important both for the update of PANS/PANDAS Research Consortium and for other more recent studies in which the recommendation of treating verified or strongly suspected ongoing bacterial infections is highly advised (21, 78), while antibiotic prophylaxis independently of ongoing infections is not supported by evidence because of a lack of robust data from literature, the impact on antimicrobial resistance and the risk of antibiotic-related adverse events.

A pilot study for penicillin prophylaxis in a small sample size of PANDAS patients has been conducted (78). However, no significant differences were observed in terms of prevention of infections in the placebo and in the treatment group, but also no differences were noticed concerning neuropsychiatric symptoms.

However, the PANS/PANDAS Research Consortium considered patients with very serious forms of PANDAS or who experienced multiple GAS-infections related exacerbations in whom it is suggested to consider a long-term GAS prophylaxis (78). In these patients, a multidisciplinary consultation with a pediatric infectious disease specialist is strongly recommended). In these selected cases, the antibiotic schedule suggested is intramuscular benzathine benzylpenicillin (600,000 UI/month; 1,200,000 UI/month for weight above 27 kg) as recommended in the guidelines for the prevention of rheumatic fever (79, 80). Another possible schedule could be oral amoxicillin (25 mg/kg once daily), although there are possible limitations in compliance with one daily administration. Although a role for azithromycin has been proposed on the basis of a study conducted in 23 pediatric patients demonstrating a reduction in neuropsychiatric exacerbations (63), the high prevalence of GAS resistance to macrolides do not permit to support recommendation of azithromycin prophylaxis for GAS prevention (81).

Great uncertainty concerns the duration of any prophylaxis scheme. At the time in which the PANS/PANDAS Research Consortium guidelines were written, anecdotal experiences with late relapses suggested a preventive regimen for at least a year or two after symptoms have completely cut down (78). Another approach for children in remission adopted by some clinicians consisted in stopping antibiotic prophylaxis during the summer period and resuming it in fall, with the attendance of child community or school (78). In case of neuropsychiatric recurrences any prophylactic antibiotic regimen must be interrupted because its ineffectiveness has been empirically demonstrated. Basically, until prospective or (even better) randomized controlled studies with robust sample sizes will be carried out, it will be difficult to express a definitive opinion on this subject.

Overall, most of the text in this statement obtained sufficient agreement during the first survey. Uncertainty was observed about antimicrobial prophylaxis, but after the review of recent literature and collective discussion on available data, panelists agreed on the above-mentioned statements.

**Statement 19.** *Before starting a prophylactic antibiotic scheme in patients with PANDAS, attention should be paid to the antibiotic resistance, weighing risks and benefits.*

The topic of antibiotic resistance is nowadays more than a concrete threat, with a continuous struggle in everyday medical practice (82). As mentioned above, there is uncertainty because conflicting results between many studies, as also stated by a recent systematic review in which three articles concerning the use of antibiotics in PANDAS were analyzed: one prospective study and one double-blind randomized controlled trial (RCT) supported the use of antibiotics, but a separate double-blind RCT showed no benefit (83). Since criteria used to diagnose PANDAS are not universally applied, extreme attention must be paid to this theme to avoid the incorrect and unwarranted use of antibiotics in order to give the best therapeutic options to the patients avoiding the risk of side effects and containing the risk of antibiotic resistance (84).

Overall, all the text in this statement obtained sufficient agreement during the first survey and was confirmed in the second survey.

**Statement 20.** *The role of procedures such as tonsillectomy and adenoidectomy in reducing the neuropsychiatric symptoms associated with PANS or PANDAS is not supported by evidence.*

The possible role of the surgical management of PANS and PANDAS has been evaluated in some studies with no evidence for possible benefits. Pavone et al. examined the impact of surgery on remission and/or modifications of the clinical course of children with PANDAS, including the severity of OCD and tics (84). The authors recruited 120 patients with PANDAS and divided them into surgical (patients who underwent tonsillectomy or adenotonsillectomy) and non-surgical groups. The follow-up showed that surgery had no impact on the clinical severity of the neuropsychiatric symptoms in these children. The authors therefore concluded that surgery should not be considered as a treatment option for this disorder, unless more significant elements suggesting the need for surgery are present (e.g. chronic inflammation or obstructive sleep apneas) (84). Similar results were obtained in the review conducted by Windfuhr (85). This review examined the benefits of tonsillectomy in PANDAS patients. Some case reports suggested a possible role for this procedure, although the examined case series did not obtain the same results. The author concluded that the benefit which seems to derive from tonsillectomy in PANDAS patients in the case reports may be related to the post-operative medications that were administered and not to the surgical procedure itself (85).

Overall, all the text in this statement 46 obtained sufficient agreement during the first survey and was confirmed in the second survey.

**Statement 21.** *There is scarce evidence on the efficacy of steroidal and non-steroidal anti-inflammatory therapy in reducing the severity of neuropsychiatric symptoms in PANS and PANDAS patients.*

The management of the neuropsychiatric symptoms associated with PANS and PANDAS frequently includes the use of anti-inflammatory therapy, both steroidal and non-steroidal. However, this habit is not supported by evidence deriving from RCT and should therefore be more deeply investigated. Brown et al. examined the efficacy of oral corticosteroids administered in short (4–5 days) or long (5 days–8 weeks) duration regimens on the symptomatic flares in the PANS or PANDAS patients (86). The results of the study showed that the treatment with oral corticosteroids shortened the duration of flares and of the initial PANS and PANDAS episodes, especially when given close to the onset of symptoms. The longer steroid courses had a more persistent impact on the improvement of symptoms. The authors, however, highlighted the need for double-blind placebo controlled RCT on this topic to formally assess the efficacy of this type of treatment. The same group also evaluated the efficacy of non-steroid anti-inflammatory drugs (NSAIDS) on reducing the impact of PANS and PANDAS symptomatic flares (86). The drugs which were used in the study included Naproxen, Ibuprofen, Sulindac and Celecoxib. The study found out that the use of NSAIDS was associated to a significantly shortened duration of flares, also when the NSAIDS were used as prophylactic agents in maintenance therapy. Similarly to their previous study, the authors concluded that randomized placebo-controlled trials are needed to strengthen their recommendations (87).

Overall, all the text in this statement obtained sufficient agreement during the first survey and was confirmed in the second survey.

**Statement 22.** *Although the use of intravenous immunoglobulins (IVIG) and plasma exchange in patients with PANS or PANDAS may have some benefit during exacerbations and recurrences, their real clinical benefit is controversial.*

IVIG and plasma exchange are therapies which are usually implied in severe cases of inflammatory conditions. Some studies have evaluated their potential use in PANS and PANDAS cases (21). The use of this type of treatments may have some benefit in PANS and PANDAS patients during exacerbations and recurrences; however, the potential risk of complications should always be taken into account. The studies on the topic in the literature showed that side effects when employing IVIG and plasma exchange for the treatment of PANDAS are frequent, even though they are usually mild (e.g. headache, fever, pallor, dizziness, nausea, vomiting) (83). Pfeiffer et al. published a clinical guidance for the diagnosis and management of PANS within the Nordic countries, which was elaborated by a working group consisting of pediatric neurologists, psychiatrists and psychologists from Denmark, Sweden and Great Britain (88). Their indications for the use of IVIG in PANS patients state that this treatment should only be implied in children who are severely affected and if there is no effect by steroid therapy or if this therapy is not feasible. The use of IVIG should be in any case discussed at least within the multidisciplinary team. The proposed treatment protocol is a boost with IVIG 2 g/kg over 2 days and thereafter every month (1 g/kg over two consecutive days) for 3 months, with a possibility of extension to a maximum of 6 months (88). The authors only indicate plasma exchange in patients with autoimmune encephalitis.

A prospective, multicenter, randomized, double-blind, parallel group, placebo-controlled, phase III superiority study with three infusion cycles of IVIG or placebo administered over 2 days every three weeks for a total of nine weeks, with an additional double-blind, crossover safety and efficacy follow-up phase of three infusion cycles of Panzyga or placebo administered over 2 days every three weeks for a total of nine weeks.

Regarding plasma exchange, it offers a promising intervention for managing acute exacerbations in PANS and PANDAS, contributing to improved patient outcomes and quality of life. The main problems in pediatrics, which would require further studies for standardization, are those relating to the type of vessel to be cannulated, the need for sedation to carry out the procedure and the schedule of execution of the sessions (e.g. number of sessions, days apart between one session and another).

Overall, all the text in this statement obtained sufficient agreement during the first survey and was confirmed in the second survey.

**Statement 23.** *The first-line therapies with the highest efficacy in children with PANS and PANDAS with acute onset of psychiatric symptoms include Cognitive-Behavioral Therapy (CBT) and Selective Serotonin-Reuptake Inhibitors (SSRI). CBT and other forms of psychotherapy have an important role especially in reducing the obsessive-compulsive neuro-psychiatric symptoms in patients with PANS or PANDAS. Parents of patients with PANS/PANDAS may benefit from CBT type psychotherapy in order to ameliorate the psychological distress associated with the condition of their children.*

The psychiatric symptoms associated with PANS and PANDAS frequently include OCD (87). When not associated with PANS/PANDAS, this type of symptoms is generally firstly treated with psychiatric interventions, both pharmacological and psychotherapy. The available evidence suggests that these interventions may also be effective in children affected by PANS or PANDAS (89). In the review performed by Dop et al. about the immunological, clinical, microbiological and therapeutic aspects of PANS and PANDAS, the authors stated that the psychiatric symptoms in these patients are usually treated with CBT and that the OCD generally respond well to SSRI at small doses (90). However, the authors also recognized that some severe cases may not respond completely to this type of therapy. Windfuhr et al. suggested the use of psychiatric medications and interventions including CBT and SSRIs in children presenting with PANS or PANDAS, as these therapies are usually effective in childhood OCDs (85). Farhood et al. suggested the use of CBT for the management of PANS and PANDAS symptoms, also due to the minimal risks associated with this therapy (83). Also, Pfeiffer et al. highlighted the importance of the access to psychological and psychiatric assessment for children with PANS or PANDAS (88). They considered this assessment fundamental in order to set up a correct psycho-education and psychotherapy for the child. It is, however, fundamental to consider the peculiarity of the drug-tolerability profile of the psychiatric medications in PANS and PANDAS patients. Indeed, Thienemann et al. showed that, in their clinical practice, the use of antidepressant medications starting at lower doses (roughly ¼ of the suggested starting dose) with a slow upward titration, was well tolerated and had a considerable benefit on children with PANS and PANDAS (91).

Interestingly, despite the generally accepted efficacy of CBT for the management of the symptoms associated with PANS and PANDAS, the studies showed that the patients’ and parents’ satisfaction was generally higher with other therapies, especially antibiotics and IVIG. This is suggested for instance in the study by Hesselmark et al. (92). The CBT approach may also benefit the parents of children with PANS or PANDAS (as with other medical conditions) in dealing with the psychological stress associated with the disease of their children (92). This is particularly relevant because of PANS and PANDAS relapsing nature and because of the traumatic nature of many of the treatments commonly used. Only few pilot studies investigated this approach for parents. One example is reported in the study by Ellerkamp et al., in which the authors examined the use of brief group therapy intervention based on trauma-focused CBT for parents of children with PANS and PANDAS (93). Ten parents participated in the study. In the end, the intervention was perceived as useful and satisfactory by the parents, especially considering its positive effects on symptoms of stress and depression. The parents demonstrated more active coping and acceptance behavior after the CBT sessions (93).

Overall, most of the text in this statement obtained sufficient agreement during the first survey. Uncertainty was observed on the use of psychiatric drugs for their potential adverse events and a possible scarce handling in pediatric age, but after the review of recent literature and collective discussion on available data, panelists agreed on its employment alongside with a well acknowledged role of CBT and familiar psychotherapy.

**Statement 24.** *Dopaminergic agents may be useful in the treatment of choreiform movements and aggressivity in some children with PANS or PANDAS.*

As stated before, a psychiatric assessment and psychiatric interventions are fundamental when taking care of children with PANS or PANDAS. Besides CBT and the use of antidepressants, some antipsychotic drugs (usually dopaminergic agents) may be indicated for the treatment of PANS and PANDAS symptoms (94). Risperidone is one of the most commonly used drugs for this specific indication. The available literature suggesting the use of risperidone for the control of choreiform symptoms and aggressivity in these children mostly comes from case-reports, while prospective studies and literature reviews are currently lacking (94).

Overall, all the text in this statement obtained sufficient agreement during the first survey and was confirmed in the second survey.

**Statement 25.** *Children with OCD, tics and other neuropsychiatric disorders must receive a specific neuropsychiatric treatment for these disorders, independently from the demonstration of a recent GAS infection. The treatment of the neuropsychiatric symptoms must not be delayed while waiting for the definitive diagnosis of PANS and PANDAS.*

When a child presents to medical attention with OCD, psychiatric symptoms, chorea, psychosis, suspected encephalopathy and/or specific neurologic signs, the neurological and psychiatric evaluation of the symptoms is of fundamental importance and has the absolute priority over the other evaluations, including the investigation of a possible case of PANS and PANDAS (85). Any delay in the correct evaluation of a neurologic or psychiatric disorder may indeed have very severe consequences on the health of the child. The proposed algorithms for the management of possible PANS or PANDAS cases highlighted the importance of a precocious appropriate multidisciplinary evaluation if symptoms of particular concern are present (85).

Overall, all the text in this statement obtained sufficient agreement during the first survey and was confirmed in the second survey.

**Statement 26.** *The available evidence supporting the role of probiotics in preventing PANS or PANDAS relapses is insufficient.*

In recent years the attention of the medical community towards probiotics has highly increased, mainly due to their potential beneficial effects on many diseases and the importance of the microbiota in the homeostasis of the human body, especially concerning the gut-brain axis (95, 96). Therefore, probiotics have been included in the management of some cases of PANS and PANDAS both for the control of symptoms and the reduction of relapses. The literature evidence, however, does not support this practice as there are no studies supporting the role of probiotics in PANS or PANDAS. The PANS/PANDAS Consortium clearly stated that there is no available clinical evidence that probiotics affect the neuro-psychiatric symptomatology in these patients (78). The authors underlined the fact that this is an area of ongoing research. The only clear role of certain types of probiotics in PANS and PANDAS is the reduction of the diarrhea which may be associated with the antimicrobial treatment frequently used in this condition (97).

Overall, all the text in this statement obtained sufficient agreement during the first survey and was confirmed in the second survey.

**Statement 27.** *In the event of a confirmed GAS infection accompanied by a sudden onset of neuropsychiatric symptoms, the first line of treatment should be the standard regimen for eradicating streptococcal pharyngitis: Amoxicillin, administered at a dosage of 50 mg/kg/day divided into two doses for 6-10 days. Using antibiotics other than Amoxicillin may contribute to increased antibiotic resistance. If neuropsychiatric symptoms relapse without evidence of a GAS infection, these symptoms should not be treated with antibiotics.*

The correct use of antibiotics is of fundamental importance especially in the present time considering the increasing prevalence of antibiotic resistance worldwide (82). PANS and PANDAS are conditions which are frequently managed with an antimicrobial substance at least once in the disease course. Guidelines for the management of acute pediatric pharyngitis clearly state that the treatment of choice for GAS pharyngitis in children consists of Amoxicillin administered orally at 50 mg/kg per day in 2 or 3 doses for 6-10 days (79, 98). In many cases of PANDAS antibiotics are used incorrectly, for instance when these drugs are used as prophylaxis or when molecules different from Amoxicillin are administered in absence of a recognized allergy to Amoxicillin. The literature evidence highlighted the importance of limiting the antibiotic treatments of PANDAS in case of evidence of GAS infection in symptomatic patients (21). Children who present with psychiatric symptoms in whom GAS infection has been excluded should not receive antibiotics (99).

Overall, all the text in this statement obtained sufficient agreement during the first survey and was confirmed in the second survey.

### 3.4 Follow-up

**Statement 28.** *Approximately one third of patients with PANS or PANDAS have a chronic-progressive course requiring further treatment, while two thirds have a relapsing-remitting course. During follow-up, the most commonly reported symptoms of relapse are OCD and tics. Unrecognized and untreated exacerbations of PANS and PANDAS appear to increase the likelihood of obsessive-compulsive and tic manifestations during adulthood. Environmental factors, such as psychosocial stress, may contribute to the perpetuation of the pathology.*

The typical course of PANS and PANDAS involves an acute onset, which is necessary for diagnosis, and a subsequent variable progression or a complete remission after onset or a remitting course with exacerbations and remissions or a chronic progressive course over time (2, 21, 100). A ‘flare’, i.e. an exacerbation of PANS or PANDAS symptoms over a period of at least 24 hours, is included in the diagnostic criteria, because it is present in the original definition of the pathology.

Available data from longitudinal studies of PANS and PANDAS patients suggested that approximately one third of patients have a chronic-progressive course requiring further treatment, while two thirds have a relapsing-remitting course. Leon et al. followed 33 patients with PANDAS and during follow-up time, approximately 72% of patients presented with at least one exacerbation of symptoms but only 12% presented with clinically significant OCD and 9% with a chronic-progressive course (17, 100). In a prospective study conducted at the Stanford PANS Clinic, patients with PANS symptoms onset criteria (i.e., with symptoms criteria and having an ‘‘abrupt’’ onset) were matched with the non-PANS cohort (i.e., those with symptoms criteria but not having an ‘‘abrupt’’ onset) (17). In 40% of patients the onset of psychiatric symptoms occurred in less than 3 days (acute), in 31% of cases from 3 days to 8 weeks (sub-acute) and in 29% of cases in more than 8 weeks (insidious). Overall, 89% of patients (both PANS and non-PANS patients) had a relapsing/remitting course but 74% generally returned to baseline after relapses. Two patients presented a progressive course: one with lupus-like pathology and the other one with autoimmune encephalitis with non-specific autoimmune markers and responding to high doses of steroids. Both of these two patients returned to their baseline following immunosuppressive therapy. Instead, three patients showed a chronic course (two of them with choreic movements and one with aggressive gesture) (17).

A Swedish study defines a “flare’’ as an exacerbation of PANS-related symptoms and/or loss of function for more than 4 days (16). Authors followed-up 34 patients diagnosed with PANS for 2 to 5 years (median 3.3 years): two patients had a ‘remitting course’ without PANS symptoms for at least 1 year, 20 patients had a ‘relapsing-remitting course’ with at least one exacerbation in the last year but have been >50% of the time in remission and 12 patients had exacerbations more than 50% of the time (‘chronic/progressive course’) (16). During the follow-up period, the most frequently reported psychiatric and somatic symptoms were OCDs (62%) and tics (50%). Less frequently reported were anxiety (35%), hyperactivity/impulsivity (35%), behavioral difficulties (32%), sleep disorders (29%), depression (29%), and tiredness/fatigue (29%). In contrast, among the somatic symptoms, 36% of patients complained of abnormalities in somatic assessment, 27% of skin problems (i.e., abrasion, eczema and psoriasis) and less frequently otitis and tonsillitis. However, although complete remission was rare, significant improvement in symptoms was observed at follow-up, with only 15% of the patients having clinically significant OCD symptoms, making the use of drugs such as SSRIs and antipsychotics relatively uncommon (16). In fact, the median score at the Children’s Yale Bronwn Obsessive Compulsive Scale (CY-BOCS), the gold standard for measuring the severity of OCD symptoms, was 8 and only 15% of patients scored above 15, i.e. the minimum severity score in clinical trials for OCDs. For tics, the Yale Global Tics Severity Scale (YGTSS) has a range of values from 0 to 65, and during follow-up patients reported a mean of 4.5, with only 2 patients scoring above 30. Therefore, the long-term prognosis of patients with PANS/PANDAS was generally good with a significant clinical improvement, but more than one third of the patients showed chronic progression and continued to show debilitating symptoms years after onset, requiring further pharmacological and psychological treatment (16).

Factors that may contribute to disease perpetuation include adverse environmental conditions, such as chronic psychosocial stress, which may exacerbate the long-term consequences of GAS exposure by promoting central immunomodulatory and oxidative stress (24). Therefore, it is certainly important to be able to identify and treat symptomatic flares promptly, as unrecognized and untreated exacerbations appear to increase the likelihood of OCD and tic manifestations during adulthood and the natural history of untreated OCD disorders is to worsen in terms of increasingly structured OCD thoughts (16). Many patients recover completely from PANS and PANDAS; therefore, it is important to treat symptoms aggressively and decrease psychosocial stressors.

Overall, most of the text in this statement obtained sufficient agreement during the first survey. Uncertainty was observed on the percentage of PANS and PANDAS patients with a chronic/progressive course, but after the review of recent literature and collective discussion on available epidemiological data, panelists agreed on the above-mentioned statements.

***Statement 29.*** *During a 5-year follow-up, about one-third of PANS and PANDAS patients may receive a new diagnosis of neuropsychiatric pathology, while one-third may develop an autoimmune or inflammatory disease, so a multidisciplinary follow-up is recommended.*

During the follow-up period, PANS and PANDAS patients may receive a diagnosis of a new pathology, in 1/3 of cases this may be a neuropsychiatric pathology and in 1/3 of cases an autoimmune or autoinflammatory pathology (89). It is important to be aware of this possibility in order to be able to recognize them promptly. In the longitudinal study conducted by Leon et al., the 33% of the 33 patients followed had received a new diagnosis of a neuropsychiatric disorder, mainly represented by attention deficit hyperactivity disorder (ADHD) (100). In the Swedish longitudinal study that followed 34 PANS/PANDAS patients, two subjects already had a previous neuropsychiatric disorder, i.e. Autism Spectrum Disorders (ASD), but 13 patients (38%) received a new diagnosis. Of these patients, nine showed ADHD (26%), three ASD (9%) and one intellectual disability (3%) (100). This highlights the importance of routine neuropsychiatric assessment in these patients.

Recognition and correct diagnosis of these symptoms will help in the implementation of therapeutic interventions for specific problems, in addition to intervention for PANS. Clinician, parents and child ratings are essential to assess and quantify cardinal symptoms of PANS, such as OCD, depression, conduct disorder and anxiety, and to identify new neuropsychiatric symptoms promptly. De Visscher et al. proposed a rating scale protocol (101). The clinician-rated scales are: Children’s Global Assessment Scale (CGAS), Clinical Global Impression–Severity Scale (CGI–S), Children’s Yale-Brown Obsessive Compulsive Scale (CY-BOCS) and Yale Global Tic Severity Scale (YGTSS). The parent-rated scales are: CGI-S, KIDSCREEN – 10, Short Moods and Feelings Questionnaire-Parent version (SMFQ-P), Separation Anxiety Avoidance Inventory-Parent version (SAAI-P), Autism Spectrum Quotient Child/Adolescent version-10 (AQ-10) and Swanson, Nolan and Pelham scale-IV (SNAP-IV). Child-rated scales are: CGI-S, KIDSCREEN-10, Short Moods and Feelings Questionnaire-Child version (SMFQ-C) and Insomnia Severity Index Child/Adolescent version (ISI-C) (101).

In terms of autoimmune or autoinflammatory conditions, the most frequent are severe atopic eczema, various food allergies, severe asthma, post-infectious arthritis, celiac disease, autoimmune thyroiditis, Henoch-Schonlein purpura and others (16).

Given the wide range of pathologies that can occur, it is advisable to perform a multidisciplinary follow-up in order to be able to detect the onset of new pathologies as soon as possible, also with the help of blood tests that can help us monitor the course of the pathology.

Overall, all the text in this statement obtained sufficient agreement during the first survey and was confirmed in the second survey.

**Statement 30.** *Hematochemical examinations of clinical utility at the onset of the disease and of the exacerbations in patients with PANS and PANDAS include: blood count with formula, C reactive protein (CPR), erythrocyte sedimentation rate (ESR), TAS, anti-DNase antibodies, serology for M. pneumoniae, vitamin D serum level, hepato-renal and thyroid function, protein foresis, IgA, IgM, IgG, antinuclear antibodies (ANAs) and interleukin (IL)-6.*

Blood tests are recommended not only at the onset of the disease, but also during the follow-up period, especially at the onset of exacerbations (17). In a 5-year prospective follow-up study of 34 PANDAS patients, the following laboratory abnormalities were found: 78% of patients had abnormal blood count, 74% altered protein phoresis, 72% reduced complement, 19% TPO antibody elevation, 15% TSH abnormalities, 11% low T4, 7% ANA positivity, 4% low IgG, 41% IgG subclass deficiency. IL-1 beta was increased in 36%, IL-10 in 20% and TNF-alpha in 19% (16). These laboratory tests are also useful for early monitoring and diagnosis of new pathologies that may occur during follow-up.

Overall, all the text in this statement obtained sufficient agreement during the first survey and was confirmed in the second survey.

**Statement 31.** *It is important to monitor infections closely during follow-up, as any intercurrent infection may be a trigger for an exacerbation. Therefore, in the event of an exacerbation, the following should be done: patient history collection, physical examination to detect any infectious focus, microbiological exams based on suspected infectious foci, throat swab, monitoring of the patient’s contacts. All vaccinations are recommended, including annual influenza vaccination.*

Since any intercurrent infection may cause a symptomatic flare, a close monitoring of the patient and prompt appropriate therapy is required. The PANS Research Consortium guidelines summarized a practical approach to infection management with the aim to identify any infectious focus and exclude co-existing causes of infection (78). A detailed anamnesis should be taken, paying particular attention to exposure to contagious ill contacts, persistent cough in the past 3-4 weeks, any respiratory or febrile illness. The physical examination should pay particular attention to infectious foci, including throat, skin, oral cavity, lymphatic system, perianal region and others, and then microbiological exams should be performed on the basis of the history and physical examination. Microbiological exams include throat swab for GAS, serum TAS, anti-DNase antibodies, nasopharyngeal or throat swab for *M. pneumoniae* PCR with specific serology. Perineal, oral and skin culture that should be obtained from suspected infectious sites. Once an infection has been confirmed by culture tests, appropriate therapy must be given. It is sometimes difficult to establish a clear temporal relationship between infection and exacerbation of symptoms, as neuropsychiatric symptoms may appear 3-4 days before the infectious symptoms; therefore, it is recommended that these measures be taken in the presence of a flare, even in the absence of infectious symptoms. Close monitoring for infection is strongly recommended in close contacts of any patient; indeed, flares of neuropsychiatric symptoms have been reported after contact with a GAS-infected family member, even though the PANS and PANDAS patient had no evidence of a clear active infection. This is seen as part of the prevention of GAS reinfection, which could prolong disease remission periods and reduce exacerbations (797). For family members of patients, it is also recommended to pay attention to symptoms suggestive of GAS infection even in unaffected siblings aged 3 to 12 years, not only to protect the index patient, but also because they have an increased genetic risk of developing PANDAS (102, 103).

Additionally, standard childhood vaccines are strongly recommended for PANS and PANDAS patients and also to all close contacts (104). Flares or exacerbations after routine childhood vaccinations, including pneumococcal vaccine, are infrequent or at most temporary and can be treated with non-steroidal anti-inflammatory drugs. Annual influenza vaccination is recommended, as well as preventive measures, such as hands washing, avoidance of exposed contacts, correct coughing technique, vigilance of influenza symptoms in patients and their close contacts during the community epidemic season, in order to make an early diagnosis and start treatment promptly (105).

Overall, all the text in this statement obtained sufficient agreement during the first survey and was confirmed in the second survey. Uncertainty was at first noticed by panelists concerning a close monitoring for infections as triggers of PANS and PANDAS exacerbations, but after a collective discussion based on current literature, panelists agreed also on the importance of this aspect.

**Statement 32.** *Follow-up visits in PANS and PANDAS patients should be performed every 2 weeks during exacerbation and every 12 weeks during periods of remission.*

The timing of follow-up visits is important, but there is no clear guidance in the literature on the timing, which could be individualized according to each patient’s disease ‘phenotype’, based on the severity of exacerbation episodes and symptomatology. A complete visit with physical examination could be done approximately every 12 weeks during the periods of remission, possibly using telemedicine tools (106). However, a closer monitoring (depending on the severity of symptoms) during an exacerbation is recommended to evaluate the available therapeutic options (107).

Overall, all the text in this statement obtained sufficient agreement during the first survey and was confirmed in the second survey.

**Statement 33.** *Performing polysomnography (PSG) may be useful to identify patients with sleep disturbance that adversely affect the clinical course of PANS and PANDAS.*

Up to 80% of patients with PANS and PANDAS complain of sleep disturbances, often present from the onset of neuropsychiatric symptoms and remitting after the severe phase or during the course of the illness. The most commonly reported disorders are parasomnias (i.e., somnambulism, nocturnal pavor, nightmares), early awakening (i.e., terminal insomnia), difficulty falling asleep or maintaining sleep (i.e., early or middle insomnia), rapid eye movement (REM) sleep disorders (i.e., REM sleep without atonia [RSWA]) and sleep movement disorders (i.e., periodic limb movement disorder [PLMD]) (107). In their prospective study, Gagliano et al. followed 23 PANS patients who had been off medication, including natural substances such as melatonin, for at least 6 weeks (107). At the time of enrolment, 9 patients out of 23 were in the acute phase of the disease, 14 out of 23 had a chronic course or were in a disease remission phase. Overall, 73.9% of the patients showed abnormalities at polysomnography (PSG): 47% ineffective sleep, 58.8% fragmented sleep, 64.7% RSWA and 47.1% PLMD. Respiratory parameters were only available for 78.2% of the patients: of these, 77.8% presented snoring and 33% pediatric sleep apnoea syndrome (OSAS), 4 had a mild apnoea/hypopnoea index (AHI) and 2 a severe AHI. The latter 2 patients underwent adenotonsillectomy surgery and reported an improvement in both apnoeas and PANS symptoms (107). Another retrospective study conducted in the Stanford pediatric clinic, followed 9 PANS patients (108). Insomnia was the most reported sleep problem, with a low average sleep duration and a decrease in sleep efficiency of 77%. PLMI was elevated in REM sleep in 7 patients and RSWA was reported in 2 patients. Therefore, the onset of PLMD is possible several years after diagnosis and treatment, and especially in cases where there was a delay in treatment or in cases of PANS with a chronic–remittent course (108). PANS symptoms, such as OCDs and tics, and sleep disorders have elements in common, regulating both dysfunction, attentional alertness and motor disturbances. This could be explained by the dopaminergic system that comes into play in the regulation of different functions, such as cognitive and motor behavior and sleep-wake rhythm (109). Furthermore, microstructural abnormalities in encephalic structures that are important for the sleep-wake rhythm (i.e., the basal ganglia, thalamus and amygdala) have been detected by MRI (11). For this reason, a PSG is recommended in PANS and PANDAS patients who complain of sleep disturbances.

Overall, uncertainty was noticed about the text in this statement since few data are available about the quality of sleep in PANS and PANDAS patients. After collegial discussion and revision of literature, sufficient agreement was reached in the second survey.

**Statement 34.** *During follow-up of PANS and PANDAS patients, a cardiological evaluation with ECG and echocardiography is useful to rule out cardiological abnormalities.*

A history of GAS infection is a risk factor of development of sequelae caused by molecular mimicry with production of cross-reacting antibodies with certain brain areas, such as basal ganglia and heart valves (10, 110). Production of anti-neuronal antibodies and antibodies against heart valves may also occur in PANS and PANDAS, although the cardiac involvement has been little investigated in past literature (30, 111). Murciano et al. has conducted a cardiological study on 30 patients (aged 6-15 years) with diagnosis of PANS and PANDAS, including a clinical examination, ECG and echocardiography (112). Results showed that 17 children had a cardiac systolic murmur under auscultation and one an inconsistent doubling of II cardiac tone. Echocardiography showed mild mitral valve insufficiency in 5 patients, whereas ECG showed sharp T waves in V1 and V2 derivations in one patient (99). These outcomes confirmed the higher prevalence of heart involvement in PANS and PANDAS population, but unexpectedly there was no association between these heart findings and antistreptolysin-O titers (112). Thus, even if a clear association between PANS and PANDAS and cardiological abnormalities has not been demonstrated, it may be possible. For this reason, a cardiological evaluation of patients with PANS and PANDAS is recommended during follow-up.

Overall, all the text in this statement obtained sufficient agreement during the first survey and was confirmed in the second survey.

**Statement 35.** *During exacerbations, low iron serum levels correlate with greater overall impairment, risk of associated inflammatory diseases and chronic PANS or PANDAS.*

Iron deficiency ranks among the most prevalent nutritional disorders in childhood, attributable to factors such as low dietary intake, absorption deficits or chronic diseases (113). The impact of iron deficiency on neurological development in childhood has been underscored by research, with iron deficiency observed in children with various conditions including anxiety disorders, ADHD, tics, depression, and febrile seizures (114, 115). The estimation of the body’s total iron reserves is reflected in the serum ferritin value, which also serves as an acute-phase protein.

Serum ferritin increases during infections, acting as a defense mechanism against bacterial agents and modulating inflammation (116). Despite the association between GAS infection, inflammation, and PANS or PANDAS, patients with this pathology often exhibit low ferritin levels, contrary to expectations, especially during flare exacerbations. In a prospective study, Chan et al. evaluated the iron serum level of 79 PANS and PANDAS patients, revealing that 27% had hypoferritinemia, with three-fourths of these cases occurring during exacerbations (117). Hypoferritinemia was commonly associated with chronic PANS, increased global impairment, and heightened caregiver burden. A plausible explanation for the elevated incidence of hypoferritinemia in PANS and PANDAS patients, particularly during a PANS flare, could be a reduced iron supply from the gut and macrophages due to increased hepcidin production, coupled with augmented iron release into the inflamed brain (117).

Uncertainty was initially observed on the iron serum levels in PANS and PANDAS patients. After review of recent literature and collective discussion on available data, panelists agreed on considering iron deficiency an important element to evaluate and eventually treat.

**Statement 36.** *Vitamin D prophylaxis is suggested for patients with PANS and PANDAS.*

There is evidence suggesting that vitamin D may act as a protective factor against respiratory tract infections, as several studies have demonstrated an association between low vitamin D levels and a history of recurrent infections and pharyngitis (118, 119). Jolliffe et al. conducted a systematic review of randomized controlled trials to explore the link between vitamin D supplementation and the prevention of acute respiratory infections (120). The results revealed a small but significant protective effect of vitamin D supplementation compared to a placebo in reducing the frequency of acute respiratory infections. The efficacy varied based on dosing regimen, study duration, and the age of participants at enrollment. The most significant outcome was observed with daily doses of 400-10,000 IU of vitamin D taken for 12 months (120).

Vitamin D plays a crucial signaling role in regulating innate and adaptive immune responses and immunoregulation (121). Receptors located in immune system cells enable vitamin D to regulate the activities of macrophages, dendritic cells, and other toll-like receptor-mediated actions. Additionally, vitamin D modulates the expression of cathelicidin and beta-defensin of innate immunity, both of which have chemotactic and toxin-neutralizing actions. In terms of adaptive immunity, vitamin D induces the expression of Th1 to Th2 cytokines. Furthermore, vitamin D appears to act as a natural immune modulator involved in the pathophysiology of autoimmune disorders, functioning as a down-regulator. This includes inhibiting dendritic cell differentiation and Th1 and Th17 cell responses (121). This immune modulating effect extends to the central nervous system. In multiple sclerosis, there is a hypothesis that the synergy of vitamin D with disease-modifying therapies may have a beneficial effect on inflammatory events, relapses, or pathology progression as well as a small protective effect on MRI lesions (122). The PANS/PANDAS Research Consortium suggested optimizing vitamin D levels in children with neuropsychiatric symptoms triggered by infections (78). Vitamin D supplementation is a low-cost and low-risk intervention, without the risk of promoting serious adverse events. While the optimal level of serum 25-OH vitamin D has not been precisely defined, it is currently recommended to maintain a level above 30 ng/mL (75 nmol/L) (123). This level can be achieved with a standard vitamin D3 supplement at a dosage of 800-1000 IU/day for children up to 5 years and 1000-2000 IU for those aged 6 years and above. Particular attention should be paid to individuals with risk factors for hypovitaminosis (i.e., black, Asian, or Hispanic race, excess weight) (124).

Uncertainty was initially observed about the need for vitamin D prophylaxis in PANS and PANDAS patients. After reviewing the recent literature and a collective discussion on available data, panelists agreed on optimize the vitamin D levels, especially in children with risk factors.

**Statement 37.** *CBT of children and adolescents with PANS or PANDAS and their families should also be continued during follow-up.*

While the etiology of PANS remains unclear, there is evident impairment in behavioral impulse control, as evidenced by commonly associated difficulties in emotion regulation, oppositionality, and developmental regression. This exacerbates the challenge of suppressing behavioral responses to anxiety and adapting to subjective distress (59). Drawing upon the proven efficacy of CBT for children with obsessive-compulsive symptoms (125), CBT emerged as a potentially effective treatment approach for PANS patients, particularly those with a partial response to medical therapy. In a 2015 trial, eight patients with OCD and onset compatible with PANS as well as their parents underwent CBT, consisting of a maximum of 14 sessions delivered both in-person and via webcam (126). Results indicated a notable 49% and 50% reduction in CY-BOCS scores at post-CBT and follow-up, respectively, compared to pretreatment levels. Concurrently, parental ratings of ‘internalizing problems decreased at both post-CBT and follow-up. Moreover, there was an improvement in parent-rated impairment across various domains such as home, school, and social functioning after treatment (126, 127). These findings suggested the utility of combining CBT with medical therapy to modify the course of OCD symptoms in children with PANS, particularly in cases refractory to medical treatment alone. Additionally, this combined approach provides substantial support to family members.

Overall, all the text in this statement obtained sufficient agreement during the first survey and was confirmed in the second survey.

## 4 Discussion

Our study highlights the difficulties and controversies surrounding the diagnosis and management of PANS and PANDAS in children. Establishing a temporal relationship with GAS or another pathogen is particularly challenging. The absence of a definitive diagnostic test makes it a diagnosis of exclusion, given the similarity of PANS and PANDAS symptoms to other neuropsychiatric syndromes. Additionally, there is limited evidence for a standardized therapeutic approach and the efficacy of antibiotic prophylaxis, with concerns about antibiotic resistance that pose further challenges. PANS and PANDAS is a relatively new clinical entity and there is insufficient information on long-term follow-up and management strategies for complicated cases. While some follow-up studies extend for at least five years, none cover a longer period. Multidisciplinary care involving various medical professionals is crucial.

Given the relapsing-remitting nature of the disease, the role of the primary care pediatrician is crucial during remission periods when visits to multidisciplinary specialists may decrease. The primary care pediatricians can promptly detect alarm signals based on clinical conditions observed during routine visits, alerting relevant specialists. They can also contribute to the surveillance and early diagnosis of infectious episodes by prescribing examinations to identify the involved pathogens. Also the family plays a crucial role in managing these patients, often recognizing the first warning signs of relapses and the onset of new neuropsychiatric pathologies. They also contribute to maintaining the psycho-physical well-being of patients. Providing psychological support to various family members is equally important.

Our consensus document aims to standardize the management of patients with suspected PANS or PANDAS and offer guidance to healthcare professionals. Thanks to the Delphi method, participants in the project, coming from diverse areas of pediatrics such as infectious diseases, neurology, rheumatology and immunology, successfully reached a clear agreement on various statements through active discussion. **Table 1** summarizes the 37 statements on epidemiology, diagnosis, therapy and follow-up of PANS and PANDAS. The results serve as the foundation for an evidence-based approach and for developing educational interventions to enhance the diagnosis and management of PANS and PANDAS.

**Table 1:** Recommendations on epidemiology, diagnosis, therapy and follow-up of PANS and PANDAS.

Epidemiology
**Statement 1.** *PANDAS is a term used to describe populations of children in whom symptoms of obsessive-compulsive disorder (OCD), tics or other neuropsychiatric disorders are exacerbated by GAS infection. PANDAS is considered a subset of the PANS category.* **Statement 2.** *Epidemiologically, the true incidence and prevalence of PANS and PANDAS are not known. The male gender seems to be more affected. Tics typically appear at an average age of about 6 years and OCD symptoms at about 7 years. In any case, children between the ages of 5 and 12 years would be more susceptible to developing PANDAS because of their frequent exposure to GAS and their tendency to respond to infections with more antibodies than adolescents and adults.* **Statement 3.** A *genetic predisposition to PANDAS development is suspected, although genetic testing is not currently required for diagnosis.* **Statement 4.** *PANDAS seems to be more common among children with a family history of rheumatic fever.* **Statement 5.** *It is supposed that a previous GAS infection could act as a PANDAS trigger, due to molecular mimicry mechanisms, causing an abnormal immune response. Moreover, a role of increased oxidative stress and neuroinflammation dysregulation caused by gut microbiota, has been proposed. For these reasons, immunomodulatory therapy could be considered in selected cases.*
Diagnostic Criteria
**Statement 6.** *PANDAS is defined by: 1) the presence of OCD and/or tic disorders; 2) onset in childhood between the age of 3 and the onset of puberty; 3) a clinical course characterized by sudden onset and dramatic worsening of symptoms; 4) the temporal relationship between GAS infection and onset/recurrence of symptoms; 5) the presence of neurological symptoms on physical examination during a relapse, such as motor hyperactivity (difficulty staying seated, restlessness) and choreiform movements, especially when aggravated by forced postures.* **Statement 7.** *PANS diagnostic criteria are sudden and dramatic onset of OCD and/or restrictive food intake (<48 hours) associated with concomitant presence of additional neuropsychiatric symptoms. The OCD symptoms must be severe and frequent. Additional neuropsychiatric symptoms should be at least two of the following: 1) anxiety; 2) emotional instability and/or depression; 3) irritability, aggressiveness and/or severe oppositional behaviors; 4) emotional regression; 5) deterioration of school performance (related to symptoms similar to attention deficit/hyperactivity disorder, memory deficits, cognitive changes); 6) sensory or motor abnormalities; 7) somatic signs and symptoms, including sleep disorders, enuresis or urinary frequency. Symptoms should not be explained by a known neurological or medical disorder, such as Sydenham’s chorea.* **Statement 8.** *The diagnosis of onset or exacerbation of PANDAS requires evidence of a close temporal association with GAS infection, although the time window is not yet well defined.* **Statement 9.** *Aetiological agents implicated in the development of PANS include varicella virus, Epstein-Barr virus, herpes simplex virus, Mycoplasma pneumoniae, Enterovirus, Chlamydia pneumoniae, Borrelia burgdorferi, Toxoplasma gondii, and SARS-CoV-2. The most common clinical manifestation related to PANS onset seems to be an upper respiratory tract infection, including rhinosinusitis or pharyngitis.* **Statement 10.** *The etiology of PANS is not necessarily infectious.* **Statement 11.** *The diagnosis of GAS infection in patients with PANDAS is made when a throat or skin swab (rapid antigen test or culture) is positive with high levels of antistreptolysin O/antistreptolysin titer (ASO) and anti-DNase B (ADB) antibodies.* **Statement 12.** *Diagnosis of PANS and PANDAS is a diagnosis of exclusion.* **Statement 13.** *There are currently no circulating biomarkers/autoantibodies pathognomonic for PANS or PANDAS.* **Statement 14.** *In patients suffering from PANS or PANDAS it is possible to find alterations in the EEG or neuroimaging investigations. These tests are not part of the diagnostic work-up for PANS/PANDAS but can be useful to exclude other organic pathologies.*
Therapy
**Statement 15.** *The PANS and PANDAS treatment is based on antibiotics, psychotherapeutic treatment (through drugs and/or cognitive-behavioral therapy) and –on immunomodulatory therapy in severe selected cases.* **Statement 16.** *In children with PANS or PANDAS with an identified etiological diagnosis, specific treatment of infectious disease should be recommended. Neuropsychiatric symptoms (especially the obsessive-compulsive ones) improve rapidly with an effective antibiotic treatment in eradicating the infection.* **Statement 17.** *In the case of PANS triggered by viral infections, some patients showed a good response to antivirals and to corticosteroid therapy, but there is little evidence in literature. The use of antibiotics and immunomodulatory agents is not recommended in children who present only an acute psychiatric symptomatology and where an infectious etiology has been excluded.* **Statement 18.** *Antibiotic therapies in children with PANDAS are effective in eradicating the causative pathogen during the active phase of the infection, but further evidence is needed to determine its effectiveness in terms of prophylaxis. There are no studies identifying an appropriate dosage for a possible prophylaxis schedule in patients with PANDAS: a possible antibiotic prophylaxis scheme could be represented by intramuscular penicillin benzathine 600,000 IU/month (1,200,000 IU/month for weight > 27 kg), similarly to the schemes used in the prevention of acute articular rheumatism. Another possible scheme could be oral amoxicillin (25 mg/kg once daily). The duration of one of the antibiotic prophylaxis schemes remains unknown. Antibiotic prophylaxis should be stopped if the neuropsychiatric symptoms do not improve or in case the child has a recurrence of neuropsychiatric symptoms while receiving antibiotic prophylaxis.* **Statement 19.** *Before starting a prophylactic antibiotic scheme in patients with PANDAS, attention should be paid to the antibiotic resistance, weighing risks and benefits.* **Statement 20.** *The role of procedures such as tonsillectomy and adenoidectomy in reducing the neuropsychiatric symptoms associated with PANS and PANDAS is not supported by evidence.* **Statement 21.** *There is scarce evidence on the efficacy of steroidal and non-steroidal anti-inflammatory therapy in reducing the severity of neuropsychiatric symptoms in PANS and PANDAS patients.* **Statement 22.** *Although the use of intravenous immunoglobulins (IVIG) and plasma exchange in patients with PANS or PANDAS may have some benefit during exacerbations and recurrences, their real clinical benefit is controversial.* **Statement 23.** *The first-line therapies with the highest efficacy in children with PANS or PANDAS with acute onset of psychiatric symptoms include Cognitive-Behavioral Therapy (CBT) and Selective Serotonin-Reuptake Inhibitors (SSRI). CBT and other forms of psychotherapy have an important role especially in reducing the obsessive-compulsive neuro-psychiatric symptoms in patients with PANS and PANDAS. Parents of patients with PANS or PANDAS may benefit from CBT type psychotherapy in order to ameliorate the psychological distress associated with the condition of their children.* **Statement 24.** *Dopaminergic agents may be useful in the treatment of choreiform movements and aggressivity in some children with PANS or PANDAS.* **Statement 25.** *Children with OCD, tics and other neuropsychiatric disorders must receive a specific neuropsychiatric treatment for these disorders, independently from the demonstration of a recent GAS infection. The treatment of the neuropsychiatric symptoms must not be delayed while waiting for the definitive diagnosis of PANS and PANDAS.* **Statement 26.** *The available evidence supporting the role of probiotics in preventing PANS or PANDAS relapses is insufficient.* **Statement 27.** *In the event of a confirmed GAS infection accompanied by a sudden onset of neuropsychiatric symptoms, the first line of treatment should be the standard regimen for eradicating streptococcal pharyngitis: Amoxicillin, administered at a dosage of 50 mg/kg/day divided into two doses for 6-10 days. Using antibiotics other than Amoxicillin may contribute to increased antibiotic resistance. If neuropsychiatric symptoms relapse without evidence of a GAS infection, these symptoms should not be treated with antibiotics.*
Follow-up
**Statement 28.** *Approximately one third of patients with PANS or PANDAS have a chronic-progressive course requiring further treatment, while two thirds have a relapsing-remitting course. During follow-up the most commonly reported symptoms of relapse are OCD and tics. Unrecognized and untreated exacerbations of PANS and PANDAS appear to increase the likelihood of obsessive-compulsive and tic manifestations during adulthood. Environmental factors, such as psychosocial stress, may contribute to the perpetuation of the pathology.* **Statement 29.** *During a 5-year follow-up, about one-third of PANS and PANDAS patients may receive a new diagnosis of neuropsychiatric pathology, while one-third may develop an autoimmune or inflammatory disease, so a multidisciplinary follow-up is recommended.* **Statement 30.** *Hematochemical examinations of clinical utility at the onset of the disease and of the exacerbations in patients with PANS and PANDAS include: blood count with formula, C reactive protein (CPR), erythrocyte sedimentation rate (ESR), TAS, anti-DNase antibodies, serology for M. pneumoniae, vitamin D serum level, hepato-renal and thyroid function, protein foresis, IgA, IgM, IgG, antinuclear antibodies (ANAs) and interleukin (IL)-6.* **Statement 31.***It is important to monitor infections closely during follow-up, as any intercurrent infection may be a trigger for an exacerbation. Therefore, in the event of an exacerbation, the following should be done: patient history collection, physical examination to detect any infectious focus, microbiological exams based on suspected infectious foci, throat swab, monitoring of the patient’s contacts. All vaccinations are recommended, including annual influenza vaccination.* **Statement 32.** *Follow-up visits in PANS and PANDAS patients should be performed every 2 weeks during exacerbation and every 12 weeks during periods of remission.* **Statement 33.** *Performing polysomnography may be useful to identify patients with sleep disturbance that adversely affect the clinical course of PANS and PANDAS.* **Statement 34.** *During follow-up of PANS and PANDAS patients, a cardiological evaluation with ECG and echocardiography is useful to rule out cardiological abnormalities.* **Statement 35.** *During exacerbations, low iron serum levels correlate with greater overall impairment, risk of associated inflammatory diseases and chronic PANS or PANDAS.* **Statement 36.** *Vitamin D prophylaxis is suggested for patients with PANS and PANDAS.* **Statement 37.** *Cognitive-behavioral treatment of children and adolescents with PANS or PANDAS and their families should also be continued during follow-up.*

It is important to note that this study has limitations, given that it relies on an opinion survey and achieved consensus in a collegial meeting. However, despite these limitations, the obtained results provide valuable insights due to the deep and accurate analysis of the literature and the large number of experts involved coming from different pediatric subspecialties. Another limitation is that all participants in Delphi method and consensus discussions were Italian practitioners. The standards of care in Italy may differ from those in other nations of the world, and further international studies that compare PANS and PANDAS management in different countries are needed.

## 5 Conclusions

This consensus provides shared indications on PANS and PANDAS management in pediatric age, based on the most updated literature. This work represents, in our opinion, the most complete and up-to-date information on the diagnosis of PANS and PANDAS, as well as consensus statements about several aspects of clinical care. Undoubtedly, more randomized and controlled trials are needed in the pediatric population to better define the best management, also in terms of adequate follow-up examinations and period of observation.

## Data Availability

The original contributions presented in the study are included in the article/supplementary material, further inquiries can be directed to the corresponding author/s.
